# Brazilian Dialysis Survey from 1999 to 2024: Trends in Dialysis Modalities and Funding

**DOI:** 10.34067/KID.0000001135

**Published:** 2026-01-12

**Authors:** Fabiana B. Nerbass, Helbert N. Lima, Fernanda S. Gorayeb-Polacchini, Ricardo C. Sesso, Jocemir R. Lugon

**Affiliations:** 1 Fundação Pró-Rim, Joinville, Brazil; 2 Sociedade Brasileira de Nefrologia, São Paulo, Brazil; 3 Universidade da Região de Joinville, Joinville, Brazil; 4 Hospital de Base, São José do Rio Preto, Brazil; 5 Universidade Federal de São Paulo, São Paulo, Brazil; 6 Universidade Federal Fluminense, Niterói, Brazil

**Keywords:** chronic hemodialysis, dialysis, minority health and disparities, peritoneal dialysis

## Abstract

For 25 years, the Brazilian Society of Nephrology has systematically monitored chronic dialysis care through the Brazilian Dialysis Survey, offering a unique perspective on the evolution of dialysis practices and access to treatment in a large and diverse middle-income country. Analysis of data from 1999 to 2024 reveals that hemodialysis has remained the dominant modality, representing on average 92% of treatments, while peritoneal dialysis (PD) accounted for only 8% and showed a steady decline. Regional differences have been marked: the Northeast consistently reported the lowest rates of PD, while the South maintained above-average use. Funding sources also reflect broader structural dynamics in the health system. The Unified Health System (Sistema Único de Saúde) has been the backbone of dialysis provision, covering 83% of patients over the period. Yet, the role of private health insurance has expanded, with coverage increasing from 12% in 2006–2009 to 20% in 2019–2024. Interestingly, regional comparisons show contrasting patterns, with private insurance supporting a disproportionately high share of dialysis in the North compared with the general population, while in the Southeast, the opposite scenario was observed. Taken together, these findings illustrate a landscape shaped by declining use of PD, heavy reliance on public financing, and persistent regional inequities. Beyond documenting numbers, the Brazilian Dialysis Survey highlights the need for policies that promote equity, strengthen the role of PD, and ensure sustainable access to KRTs for all Brazilians.

## Introduction

In July 2024, Brazil's population was estimated at 212.6 million, ranking seventh in the world.^1^ The prevalence of chronic dialysis is approximately twice the global estimate.^2^ Chronic KRT through dialysis has been publicly funded by the Unified Health System (Sistema Único de Saúde [SUS]) for more than three decades. Although the reimbursement rates for dialysis are relatively low compared with those in other countries, resulting not highly profitable for private providers (who own approximately 75% of dialysis units), treatment prevalence in Brazil is comparable with that observed in many high-income nations.^2^

Over the past 25 years, the Brazilian Society of Nephrology (BSN) has systematically monitored chronic dialysis care in Brazil through the annual Brazilian Dialysis Survey. This long-standing initiative provides essential epidemiologic data on patients undergoing dialysis, capturing changes in clinical practices and treatment access across the country.

During this period, the number of individuals receiving dialysis increased more than four-fold, rising from 42,695 in 1999 to an estimated 172,585 patients in 2024.^3^ These years were also marked by shifts in dialysis modalities, sources of treatment funding, and notable regional differences not yet systematically analyzed.

This study aims to describe national and regional trends in the use of hemodialysis and peritoneal dialysis (PD), as well as patterns of public and private coverage for dialysis treatments in Brazil, over a 25-year period.

## Methods

Between 1999 and 2005, all chronic dialysis centers registered with the BSN were contacted directly by telephone for a national survey. During this period, an average of 534 centers participated annually. However, participation rate data were available only for 2005, when it reached 83.4%.

From 2006 to 2024, registered centers were invited to participate through email and, in later years, also through BSN's media channels. Participation in the survey has been voluntary. Responding dialysis centers completed an online questionnaire hosted on the BSN website. On average, 46% (*n*=332) of the invited centers submitted data annually. The distribution of dialysis centers across the five geographic regions in 2024 was 47% in the Southeast, followed by 19% in the Northeast, 17% in the South, 10% in the Central-West, and 6% in the North.

Data were reported in aggregate rather than at the individual level. For this analysis, we used data from the standardized forms that dialysis centers have been routinely asked to complete each year as part of the national dialysis survey. These forms require information on the number of patients according to dialysis modality and funding source. No new data were collected specifically for this manuscript; however, this is the first time that the data have been analyzed and presented with the present focus. National-level data on dialysis modalities have been available since 1999, while regional data since 2009. Data on funding sources have been available at the national level since 2006 and at the regional level since 2010. The percentage of the general population with private health insurance was obtained from the website of the National Health Agency.^4^ For the presentation of tables, prevalence data were averaged by dialysis modality across four predefined time intervals. The first period, 1999–2008, includes only national-level. Subsequent periods were 2009–2014, 2015–2020, and 2021–2024, with the latter reflecting the introduction of hemodiafiltration, which was incorporated under the hemodialysis category. Funding data were similarly grouped into four periods: 2006–2009 (national data only), 2010–2014, 2015–2019, and 2020–2024.

### Statistical Analysis

The Mann-Kendall trend test was applied to assess monotonic trends over the whole study period. For consistency in the analysis, hemodialysis and hemodiafiltration prevalences were combined. Statistical significance was set at *P* < 0.05. Analyses were performed using Python version 3.13.1.

## Results

**Figure 1:**
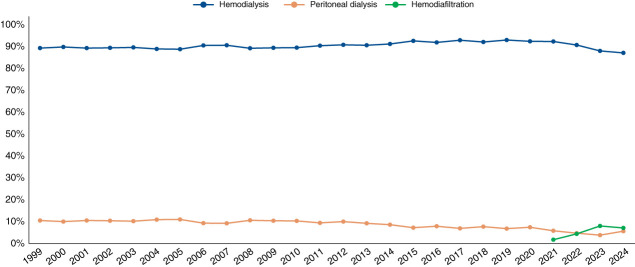
Distribution of chronic dialysis patients in Brazil according to dialysis modalities over the 25-year period.

Over the 25-year period, an average of 92% of patients was on chronic hemodialysis and 8% on PD. A significant upward trend in the prevalence of hemodialysis was observed, accompanied by a corresponding decline in the use of PD (Table 1). As shown in Figure 1, during the first 15 years (1999–2014), the distribution of dialysis modalities remained relatively stable, with hemodialysis ranging from 89% to 91% and PD from 9% to 11%. From 2015 to 2020, hemodialysis averaged 93%, while PD stabilized at 7%, a level below the historical figure. Over the past 4 years, the use of PD declined further, with only 5% of patients treated by the modality. A significantly similar trend was observed across all regions, except for the Central-West one, where no steady trend was identified. Compared with national estimates, the Northeast consistently had the lowest prevalence of PD across all periods, whereas the South consistently reported rates above the national average (Table 1).

**Table 1:** Distribution of chronic dialysis patients by dialysis modality over the years in Brazil, nationally and by geographical regions

Region	Dialysis Modalities	Average					Mann-Kendall Test
		Whole Period	1999–2008	2009–2014	2015–2020	2021–2024^a^	*P* Value for Trend^b^
Brazil	Hemodialysis	92%	90%	91%	93%	95%	<0.001
	PD	8%	10%	9%	7%	5%	
North	Hemodialysis	92%	—	87%	93%	96%	0.002
	PD	8%	—	13%	7%	4%	
Northeast	Hemodialysis	95%	—	94%	95%	98%	0.002
	PD	5%	—	6%	5%	2%	
Central-West	Hemodialysis	93%	—	94%	92%	96%	0.44
	PD	7%	—	6%	8%	4%	
Southeast	Hemodialysis	93%	—	91%	92%	94%	0.001
	PD	7%	—	9%	8%	6%	
South	Hemodialysis	90%	—	89%	90%	92%	0.005
	PD	10%	—	11%	10%	8%	

PD, peritoneal dialysis.

a Prevalence of hemodialysis+hemodiafiltration.

b Considered the whole period.

**Figure 2:**
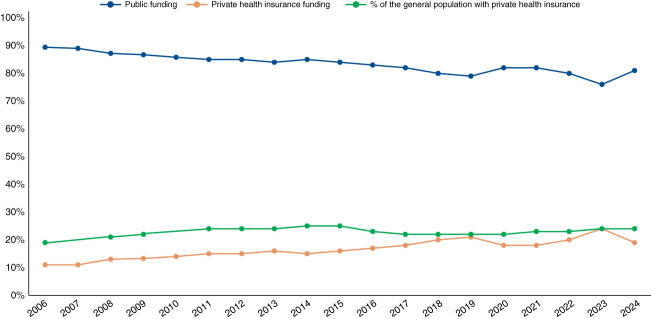
Percentage of patients undergoing dialysis with public and private health insurance funding over time, compared with the percentage of the general Brazilian population with private health insurance.

Over the 19 years of data collection on dialysis funding, an average of 83% of chronic dialysis patients was covered by the public Unified Health System, with the remaining 17% covered by private health insurance (a lower proportion than the percentage of the general Brazilian population with private health insurance during the same period, 23%; Figure 2 and Table 2).

**Table 2:** Percentage of the Brazilian general population with private health insurance and the proportion of private dialysis funding, nationally and by geographical region, over the years

Region	% with Private Health Insurance	Average					Mann-Kendall Test
		Whole Period	2006–2009	2010–2014	2015–2019	2020–2024	*P* Value for Trend^a^
Brazil	General population	23%	21%	23%	23%	23%	0.06
	Dialysis population	17%	12%	18%	20%	20%	<0.001
North	General population	10%	—	10%	10%	9%	0.02
	Dialysis population	21%	—	14%	23%	27%	0.01
Northeast	General population	12%	—	11%	12%	12%	1
	Dialysis population	12%	—	10%	14%	13%	0.02
Central-West	General population	21%	—	18%	20%	24%	0.31
	Dialysis population	23%	—	18%	23%	28%	<0.001
Southeast	General population	34%	—	37%	33%	33%	0.005
	Dialysis population	20%	—	17%	20%	23%	<0.001
South	General population	23%	—	24%	23%	23%	0.03
	Dialysis population	15%	—	14%	14%	17%	0.02

a Considering the whole period.

Over time, the gap between the proportion of patients on dialysis funded by the private system and the percentage of the general population with private insurance has decreased considerably, from 21% versus 12% in 2006–2009 to 23% versus 20% in 2019–2024. Across regions, during the whole period, the highest prevalence of public funding was observed in the Northeast (88%), followed by the South (85%), Southeast (80%), North (79%), and Central-West (77%) regions.

A significant increasing trend was observed in the prevalence of dialysis patients covered by private health insurance at the national level and across all regions (*P* for trend < 0.001). By contrast, the initial upward trend in the percentage of the population with private insurance was not sustained over time, and the overall increase was only marginally significant (*P* for trend = 0.06). Indeed, a decreasing trend was observed in the North, Southeast, and South, while prevalence remained stable in the Central-West and Northeast (Table 2).

We observed considerable regional heterogeneity when comparing the prevalence of private health insurance coverage of dialysis and the percentage of the general population with private health insurance. In the North, for instance, the average coverage over the entire period was twice as high among dialysis patients (21% versus 10%). In the Central-West, the difference was lower (23% versus 21%), and in the Northeast, no difference was observed (12% in both groups). The figures for the South and Southeast showed an opposite pattern (15% versus 23% and 20% versus 34%, respectively).

## Discussion

In this retrospective analysis of the primary dataset on chronic dialysis patients in Brazil, we observed a decline in PD utilization over the period from 1999 to 2024, whereas the proportion of patients covered by private health insurance increased steadily between 2006 and 2024 along with persistent regional disparities.

Over the 25-year observation period, hemodialysis remained the predominant dialysis modality in Brazil, accounting for an average of 92% of treatments. Although the proportion of patients on each modality was relatively stable during the first 15 years, a marked decline in PD use was observed in the most recent period, reaching an average of only 5% of users. Globally, the growing number of patients requiring dialysis has not been accompanied by equitable access to treatment, particularly in low- and middle-income countries.^5^

Our findings are consistent with recent analyses of PD use restricted to the national public health system data in Brazil, which reported a decline from 6.5% in 2014 to 4.3% in 2023.^6^ Similar to our observations, the most pronounced reductions occurred in the North and Northeast regions. Authors attribute the lack of effective public health care policies and adequate funding to incentivize centers to expand or maintain PD programs as contributing factors to the declining number dialysis centers offering this modality from 51.6% in 2014 to 37.9% in 2023.^6^ In addition, the limited number of patients undergoing PD reduces the exposure of nephrology trainees in Brazil to the modality, diminishing their confidence and competence in this regard and contributing to the perpetuation of this trend.

We were not able to analyze demographic changes exclusively within the PD population, something reported by Moura-Neto *et al.*, who found a slight increase in the median age of PD patients over the decade (from 58 to 59 years).^6^ In our historical analysis of the overall chronic dialysis population, we observed an increase in the proportion of elderly patients (>65 years) from 25% in 2006 to 37% in 2023.^7^

Another important national-level issue is the limited access to predialysis care, which consequently reduces the proportion of patients who can initiate dialysis in a planned manner. This situation hinders the use of PD as the first dialysis modality, since it requires prior catheter placement and patient training. Exceptions occur only in the few Brazilian centers that offer urgent-start PD.^8^ A recent analysis from São Paulo, the most populous Brazilian state, located in the Southeast, showed that in 2023–2024, among 8451 individuals who started dialysis with public funding (SUS), 26% had received predialysis care, 70% initiated treatment in a hospital setting, and only 5% started on PD.^9^ Additional barriers include high logistical costs for the delivery of supplies and limited patient awareness of this therapy and its potential benefits.^10^

The slight increase in PD use observed in 2024 requires confirmation in the coming years. It may reflect the effect of local public policies in some states that offered a supplementary payment to the reimbursement provided by the Unified Health System. Worldwide, approximately 11% of people on chronic dialysis are treated with PD.^11^ Globally, the pattern is heterogeneous: some countries (*e.g*., China, the United States, and Thailand) have seen increases in PD utilization, often driven by health policy incentives, cost considerations, and local infrastructure, while many European countries and Japan have experienced a decrease in the proportion of PD use. The reasons for declining prevalence in these regions include limited provider experience, concerns about technique failure, reimbursement strategies favoring hemodialysis, and logistical challenges in PD fluid supply and training.^12,13^

Our study demonstrated a substantial increase in the proportion of patients whose dialysis therapy was funded by private health insurance between 2006 and 2024. We highlight that in the past 2 years (2023 and 2024), we conducted validation exercises to verify the accuracy of registry estimates on prevalence, incidence, and funding source by obtaining parallel data from a random sample of dialysis centers nationwide. The results confirmed the consistency of the registry information: in 2023, the distribution of dialysis funding was identical in both data sources (76% public and 24% private coverage),^14^ and in 2024,^3^ the difference was of two percentage points (81% and 19% in the voluntary registry versus 79% and 21% in the random sample), supporting the reliability of the registry data.

In Brazil, dialysis is universally covered by the public health system (SUS) since January 1990, which ensures access to all patients, regardless of their socioeconomic status. Individuals with private health insurance can also receive dialysis through their private plan, which was regulated by the government in January 1999 depending on the coverage terms. The decision to use private versus public funding is therefore influenced primarily by insurance availability and contractual coverage, rather than by quality differences between systems. Most dialysis centers are accredited by both public and private payers, and patients under both schemes are often treated in the same facilities. Reimbursement rates from private insurers are generally higher than those paid by SUS, although physicians and staff typically work under similar employment arrangements regardless of the dialysis funding source.

The high heterogeneity regarding the percentage of the general population covered by private health insurance, varying from 9% in the North to 33% in the Southeast, reflects the pronounced socioeconomic disparities and unequal distribution of health care resources across Brazilian regions. Marked regional differences were identified when comparing private health insurance coverage in the general population with private financing of dialysis. These differences not only reflect the unequal distribution of private health plans across the country but also reveal distinct patterns in how patients with CKD access care in different settings.

In less affluent regions, such as the North, the proportion of dialysis patients funded by private insurance was twice the percentage of the general population with private health insurance. This may indicate that, in areas where overall coverage is low, individuals with private health insurance have greater access to dialysis treatment, suggesting a selective effect with only a small, wealthier subset of people receives private-funded care. In the Central-West, the difference between these proportions was minimal, and in the Northeast, virtually absent, indicating that in these regions, private funding of dialysis mirrors the fraction of the general population with private health insurance. On the other hand, in the more developed South and Southeast regions, the proportion of dialysis patients with private coverage was lower than the percentage of the general population with private health insurance, with the Southeast showing the largest gap. This inverse relationship could be due to restrictions on contracts, high costs, or historical limitations of coverage in health plan coverage. These disparities require further elucidation.

As study limitations, we highlight the reliance on estimates from electronic data collected through voluntary participation, the use of aggregated data at the dialysis center level, and the lack of validation of responses. Moreover, the survey includes data from about 46% of dialysis centers nationwide, which may particularly affect regions with fewer units, such as the North, and effect the representativeness of regional findings. The main strength of this study is the large and nationally representative dataset collected over 25 years, offering a unique longitudinal perspective on dialysis treatment trends in Brazil.

In conclusion, this study highlights significant shifts in dialysis modalities and funding sources in Brazil over the past 25 years, alongside pronounced regional disparities in private health insurance coverage among dialysis patients. The declining use of PD and the continued heavy reliance on the public health system to finance treatment underscore ongoing challenges within the health care system. Addressing regional inequities and expanding access to diverse dialysis options are essential steps toward improving equity and outcomes for patients with CKD nationwide. Continued monitoring and targeted policy interventions will be critical to support these goals.

## Supplementary Material

SUPPLEMENTARY MATERIAL
