# Supplementary material for: Brazilian Dialysis Survey from 1999 to 2024: Trends in Dialysis Modalities and Funding
Source: Kidney360. 2026 Jan 12;7(3):658–63. doi: 10.34067/KID.0000001135 (PMC13065121; doi:10.34067/KID.0000001135)
Supplement: SUPPLEMENTARY MATERIAL [file kidney360-7-658-s001.pdf]

ASN American Society of Nephrology | JASN Journal of the American Society of Nephrology | KIDNEY360 Accessing Our World From Every Angle | CJASN Clinical Journal of the American Society of Nephrology

ASN Journal Disclosure Form

As per ASN journal policy, I have disclosed any financial relationships or commitments I have held in the past 36 months as included below. I have listed my Current Employer below to indicate there is a relationship requiring disclosure. If no relationship exists, my Current Employer is not listed.

F. Gorayeb Polacchini has nothing to disclose.

I understand that the information above will be published within the journal article, if accepted, and that failure to comply and/or to accurately and completely report the potential financial conflicts of interest could lead to the following: 1) Prior to publication, article rejection, or 2) Post-publication, sanctions ranging from, but not limited to, issuing a correction, reporting the inaccurate information to the authors' institution, banning authors from submitting work to ASN journals for varying lengths of time, and/or retraction of the published work.

Name: Fernanda Salomao Gorayeb Polacchini
Manuscript ID: K360-2025-001124
Manuscript Title: Brazilian Dialysis Survey from 1999 to 2024 Trends in dialysis modalities and funding
Date of Completion: November 17, 2025
Disclosure Updated Date: November 17, 2025

ASN American Society of Nephrology JASN Journal of the American Society of Nephrology KIDNEY360 Accessing Our World From Every Angle CJASN Clinical Journal of the American Society of Nephrology

ASN Journal Disclosure Form

As per ASN journal policy, I have disclosed any financial relationships or commitments I have held in the past 36 months as included below. I have listed my Current Employer below to indicate there is a relationship requiring disclosure. If no relationship exists, my Current Employer is not listed.

H. Lima has nothing to disclose.

I understand that the information above will be published within the journal article, if accepted, and that failure to comply and/or to accurately and completely report the potential financial conflicts of interest could lead to the following: 1) Prior to publication, article rejection, or 2) Post-publication, sanctions ranging from, but not limited to, issuing a correction, reporting the inaccurate information to the authors' institution, banning authors from submitting work to ASN journals for varying lengths of time, and/or retraction of the published work.

Name: Helbert do Nascimento Lima
Manuscript ID: K360-2025-001124R1
Manuscript Title: Brazilian Dialysis Survey from 1999 to 2024: Trends in dialysis modalities and funding
Date of Completion: November 20, 2025
Disclosure Updated Date: November 20, 2025

ASN American Society of Nephrology | JASN Journal of the American Society of Nephrology | KIDNEY360 Accessing Our World From Every Angle | CJASN Clinical Journal of the American Society of Nephrology

ASN Journal Disclosure Form

As per ASN journal policy, I have disclosed any financial relationships or commitments I have held in the past 36 months as included below. I have listed my Current Employer below to indicate there is a relationship requiring disclosure. If no relationship exists, my Current Employer is not listed.

J. Lugon reports the following:
Employer: Universidade Federal Fluminense; and Speakers Bureau: Astrazeneca.

I understand that the information above will be published within the journal article, if accepted, and that failure to comply and/or to accurately and completely report the potential financial conflicts of interest could lead to the following: 1) Prior to publication, article rejection, or 2) Post-publication, sanctions ranging from, but not limited to, issuing a correction, reporting the inaccurate information to the authors' institution, banning authors from submitting work to ASN journals for varying lengths of time, and/or retraction of the published work.

Name: Jocemir R. Lugon
Manuscript ID: K360-2025-001124R1
Manuscript Title: Brazilian Dialysis Survey from 1999 to 2024: Trends in dialysis modalities and funding
Date of Completion: November 22, 2025
Disclosure Updated Date: November 22, 2025

ASN
American Society of Nephrology
JASN
Journal of the American Society of Nephrology
KIDNEY360
Accessing Our World From Every Angle
CJASN
Clinical Journal of the American Society of Nephrology

ASN Journal Disclosure Form

As per ASN journal policy, I have disclosed any financial relationships or commitments I have held in the past 36 months as included below. I have listed my Current Employer below to indicate there is a relationship requiring disclosure. If no relationship exists, my Current Employer is not listed.

F. Nerbass has nothing to disclose.

I understand that the information above will be published within the journal article, if accepted, and that failure to comply and/or to accurately and completely report the potential financial conflicts of interest could lead to the following: 1) Prior to publication, article rejection, or 2) Post-publication, sanctions ranging from, but not limited to, issuing a correction, reporting the inaccurate information to the authors' institution, banning authors from submitting work to ASN journals for varying lengths of time, and/or retraction of the published work.

Name: Fabiana Baggio Nerbass
Manuscript ID: K360-2025-001124
Manuscript Title: Brazilian Dialysis Survey from 1999 to 2024 Trends in dialysis modalities and funding
Date of Completion: November 17, 2025
Disclosure Updated Date: November 17, 2025

ASN Journal Disclosure Form

As per ASN journal policy, I have disclosed any financial relationships or commitments I have held in the past 36 months as included below. I have listed my Current Employer below to indicate there is a relationship requiring disclosure. If no relationship exists, my Current Employer is not listed.

R. Sesso reports the following:
Employer: Escola Paulista De Medicina, Unifesp

I understand that the information above will be published within the journal article, if accepted, and that failure to comply and/or to accurately and completely report the potential financial conflicts of interest could lead to the following: 1) Prior to publication, article rejection, or 2) Post-publication, sanctions ranging from, but not limited to, issuing a correction, reporting the inaccurate information to the authors' institution, banning authors from submitting work to ASN journals for varying lengths of time, and/or retraction of the published work.

Name: Ricardo Sesso
Manuscript ID: K360-2025-001124
Manuscript Title: Brazilian Dialysis Survey from 1999 to 2024 Trends in dialysis modalities and funding
Date of Completion: November 17, 2025
Disclosure Updated Date: November 17, 2025
